# Early detection of red palm weevil using distributed optical sensor

**DOI:** 10.1038/s41598-020-60171-7

**Published:** 2020-02-21

**Authors:** Islam Ashry, Yuan Mao, Yousef Al-Fehaid, Abdulmoneim Al-Shawaf, Mansour Al-Bagshi, Salman Al-Brahim, Tien Khee Ng, Boon S. Ooi

**Affiliations:** 1 Computer, Electrical and Mathematical Sciences and Engineering (CEMSE) division, King Abdullah University of Science and Technology (KAUST), Thuwal, 23955-6900 Kingdom of Saudi Arabia; 2 Center of Date Palms and Dates, Ministry of Environment, Water and Agriculture, Al-Hassa, Kingdom of Saudi Arabia; 3 Office of the Ministry of Environment, Water and Agriculture, Al-Hassa, Kingdom of Saudi Arabia

**Keywords:** Electrical and electronic engineering, Optical sensors

## Abstract

Red palm weevil (RPW) poses a serious threat to the cultivation of date palms. It is considered to be the most destructive epidemic pest of palms, responsible for massive economic losses worldwide. Curative methods for RPW are not difficult to apply; however, the early detection of the pest remains a great challenge. Although several detection techniques have been implemented for the early detection of RPW, none of these methods have been proven to be reliable. Here, we use an optical-fiber-distributed acoustic sensor (DAS) as a paradigm shift technology for the early detection of RPW. Our sensitive sensor shows a detection of feeding sound produced by larvae as young as 12 days, in an infested tree. In comparison with existing, commonly-used technologies, this novel sensing technique represents a cost-effective and non-invasive alternative that could provide 24-7, real-time monitoring of 1,000 palm trees or even more. It could also monitor the temperature, an essential feature to control farm fires, another major problem for the cultivation of palm trees around the world.

## Introduction

Red palm weevil (RPW) (*Rhynchophorus ferrugineus*) is a Coleopteran snout pest originating from tropical Asia^1^. In the past few decades, it has spread out to many regions worldwide, including North Africa, the Middle East, Mediterranean region, and parts of the Caribbean and Central America. This plague has wiped out many palm farms in various countries, and constitutes a severe agricultural problem^2–4^. In Gulf countries and the Middle East, $8 millions are spent, every year, only to remove infested palm trees^5^. By 2023, in Italy, Spain and France, it is expected that RPW control and loss of benefits will amount to around $225 million^5^.

There are effective techniques to heal RPW-infested palm trees^6^; however, detecting the RPW threat at an early stage (first two-to-three weeks of the weevil larvae stage) is pivotal, yet challenging^4^. When a palm tree shows visible signs of distress, it generally means that the infestation is well-advanced; at this point, it is too late to rescue the tree. There are several methods to detect this infestation^4,7–12^. For instance, trained dogs are used to smell the odor released from infested palms during the fermentation process^8^. Unfortunately, sensing such kind of odor is not an accurate, nor a selective process, because its efficiency is impacted by the presence of other volatiles. Alternatively, infested trees can be screened with a computer-based tomography system^7^. However, this technique lacks the practicality for fast and cost-effective scanning. The first detectable signals of an infested tree originate from the noise produced by the weevil larvae while consuming the core of a trunk. Therefore, the most promising early detection methods rely on using acoustic sensors^4,9–12^. Existing technologies mainly insert a sound probe into the tree trunk such that the probe records larvae sound in real-time. Unfortunately, this type of acoustic sensors is intrusive to the plant growth, and might create a nest for other insects, including RPW. Additionally, offering an acoustic sensor for each tree, along with a wireless communication interface, significantly increases the cost of the entire RPW surveillance system in order to provide constant monitoring.

Here, we report on the use of an optical-fiber-distributed acoustic sensor (DAS) and a signal processing algorithm as a robust solution for the early detection of RPW. In our design, all of the optical/electronic components including the laser and photodetector are gathered within a single unit, whereas only the fiber is wound around the palm trees to form an optical network. In our study, this new system is used to distinguish two palm trees, one healthy and one infested with ~12 days old larvae. A validation experiment is carried out in Al-Hassa city, Eastern Province, KSA, where RPW infestation is severe^13^. Compared to other existing acoustic sensors, our sensor is uniquely non-invasive, providing 24-7 monitoring, at relatively low cost, and offering wide coverage of the farming area, using only a single optical fiber cable.

## Results

**Figure 1:**
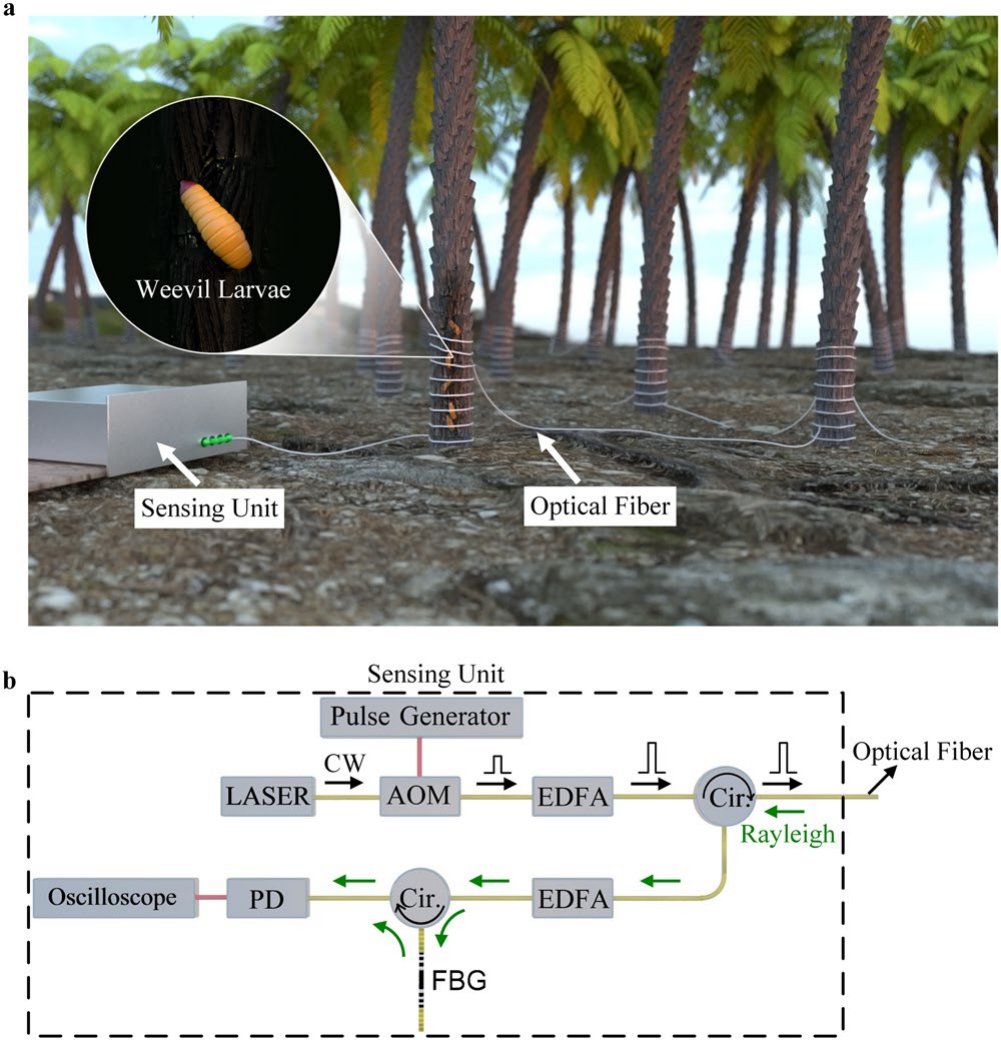
Optical fiber DAS for early detection of RPW. (**a**) Overall operation plan of new setup using an optical fiber DAS for the early detection of RPW. (**b**) Experimental setup of the optical fiber DAS: Cir., circulator.

Setup of the new operation with our optical fiber DAS is represented in Fig. 1(a). The entire optical/electronic components used to design the sensor are located within a sensing unit. This sensing unit is connected to an optical fiber that is extended throughout the palm-trees farm. The optical fiber is looped around each tree trunk, from the ground up to a ~1 m height, where the probability of finding weevil larvae is high^14^. Between trees, the optical fiber cable can be either laid down on the ground or buried in soil, based on environmental conditions. A real-time signal processing of the data provided by the sensing unit enables us to precisely and accurately identify the locations of the infested and healthy trees.

Optical fiber DAS is essentially designed using phase-sensitive optical time-domain reflectometry (Φ-OTDR)^15,16^ which has been utilized for many potential applications in the oil and gas industries^17^ and for real-time structural health monitoring^18^. Its operation principle consists in launching a train of optical pulses generated by a narrow linewidth laser into a single-mode fiber (SMF). At the fiber input port, consecutive Rayleigh backscattered traces are recorded in the time-domain. Each Rayleigh trace has a speckle-like profile because of the coherent interference of the signals reflected by scattering centers for the duration of the injected pulse^19^. In the absence of intrusion along the optical fiber, i.e., no perturbation of the refractive index, the recorded Rayleigh traces are ideally identical. When an acoustic signal is applied at a position along the fiber, such as the weevil larvae sound, the effective refractive index at this position changes, and the intrusion can be sensed by observing the intensity fluctuations of its corresponding speckle in the recorded traces. The experimental setup included within the sensing unit, shown in Fig. 1a, is illustrated schematically in Fig. 1b. A laser source generated a continuous wave (CW) light of 100 Hz linewidth, a 16dBm-optical power, and a 1535nm-operation wavelength. The laser light was modulated by an acousto-optic modulator (AOM) driven by a pulse generator to produce optical pulses of 20 kHz repetition rate and 100 ns pulse-width offering 10 m- sensing spatial resolution. Next, the power of the modulated light was amplified, using an erbium-doped-fiber-amplifier (EDFA), and then injected through a circulator into a SMF of ~1km-length. The backscattered Rayleigh signal from the SMF was recorded, using a direct detection scheme. The Rayleigh signal was initially amplified with another EDFA which amplified spontaneous emission (ASE) noise was discarded by a fiber Bragg grating (FBG). The filtered Rayleigh signal was then recorded via a photodetector (PD), sampled at 125MS/s rate using an oscilloscope, and processed to extract the sensing information.

**Figure 2:**
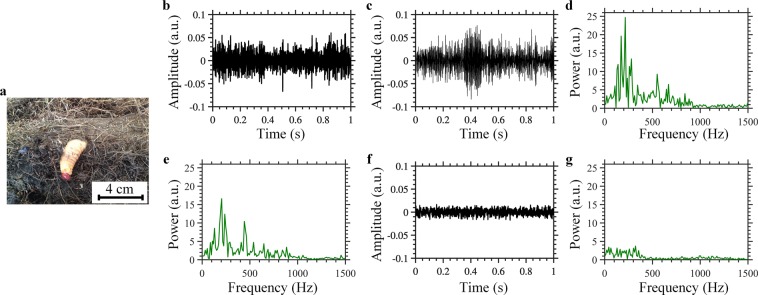
Measurements of the sound spectrum signature of the weevil larvae. (**a**) ~12 days old weevil larvae (**b**,**c**) Representative examples of time-domain signals and (**d**,**e**) corresponding power spectra of the sound emitted by the larvae, recorded via microphone. (**f**) Time-domain signal and (**g**) corresponding power spectrum of the background noise recorded via microphone.

In our experiments, we initially focused on measuring the signature of the sound spectrum of weevil larvae less than two weeks old. We selected this specific RPW life stage to test if our new sensor can detect the larvae sound at an early stage, so that the palm tree can be cured and saved. To control the age of the weevil larvae, a palm tree was artificially infested with male and female adult RPWs, and the transition from the egg- to the larvae-stage was closely monitored. In particular, we carried out our experiment using an artificially infested sample tree that included 4 pieces of ~12 days old larvae (Fig. 2a). Recognizing the sound spectrum’s signature of the larvae will facilitate discarding noises when using the optical fiber DAS to locate infested trees. To measure the sound spectrum signature, we used a commercially available voice recording microphone that we inserted into the trunk of the artificially infested palm tree that was in close proximity to the larvae. Two representative examples of the time-domain larvae sound recorded via the microphone are shown in Fig. 2b,c and their corresponding power spectra are represented in Fig. 2d,e. As shown in Fig. 2d,e, the sound from the larvae exhibits frequencies that are mostly below ~800 Hz, and may also include some environmental noise signals. Fig. 2f,g respectively show an example of time-domain signal and power spectrum of the background noise recorded via microphone, without the presence of larvae. It is worth mentioning that both the larvae sound and background noise were recorded exactly under the same setting parameters for the microphone; therefore, their signal strengths in the time and frequency domains shown in Fig. 2b–g are comparable. In contrast to the sound emitted by the larvae, the noise signal is weaker with almost flat fluctuations in the time-domain, and its power is roughly equally distributed among low frequencies (below ~400 Hz). The power spectra results of the larvae’s sound and the background noise are in good agreement with the emulation experiments performed in some of our previous work^20^.

**Figure 3:**
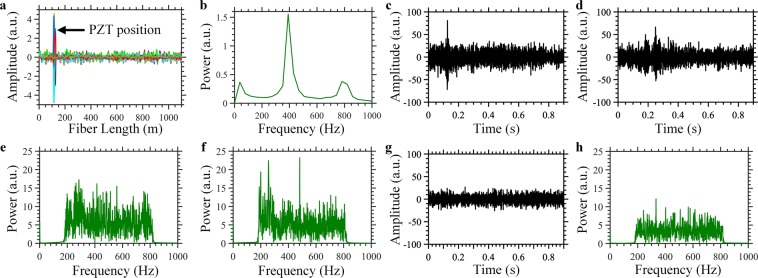
Recording weevil larvae sound with optical fiber DAS. (**a**) Position information and (**b**) power spectrum of the 400 Hz vibration event produced by a PZT cylinder. (**c**,**d**) Representative examples of time-domain signals and (**e**,**f**) their corresponding power spectra of the larvae sound recorded using the optical fiber DAS. (**g**) Time-domain signal and (**h**) its corresponding power spectrum of the background noise recorded via the optical fiber DAS.

Before starting to use our DAS to record the sound of weevil larvae, we calibrated it to make sure it can accurately find a location, precisely, along the fiber subjected to acoustic signals, and that it can also determine the frequencies of the signals for each location. For calibration purposes, at ~110 m-distance from the SMF input facet, we wound a 10 m-fiber section around a piezoelectric transducer (PZT) cylinder, such that the PZT cylinder acted as a vibration source. PZT cylinders are generally used for the calibration of optical fiber DAS because their vibrational amplitude and frequency can be predetermined using a driven-function generator^15^. As described in ref. ^19^, the location of the vibration, along the optical fiber, can be determined by subtracting the time-domain Rayleigh traces generated when injecting pulses into the optical fiber. Once the location has been found and identified, a Fourier transform is applied on the Rayleigh traces, at this precise location, in order to determine the components of the signal’s frequency. Fig. 3a,b respectively show the location’s information and the power spectrum of a 400 Hz PZT vibration event. The results obtained in this study confirm the ability of our optical fiber DAS to precisely determine the location of the PZT vibration, together with its frequencies. High-order harmonics appear at 800 Hz because of the nonlinearity of the direct detection scheme (Fig. 3b). It is worth mentioning that the aim of the PZT experiment is just to calibrate our system and to prove the developed DAS can locate vibration position and frequency, before proceeding to the RPW detection experiment.

**Figure 4:**
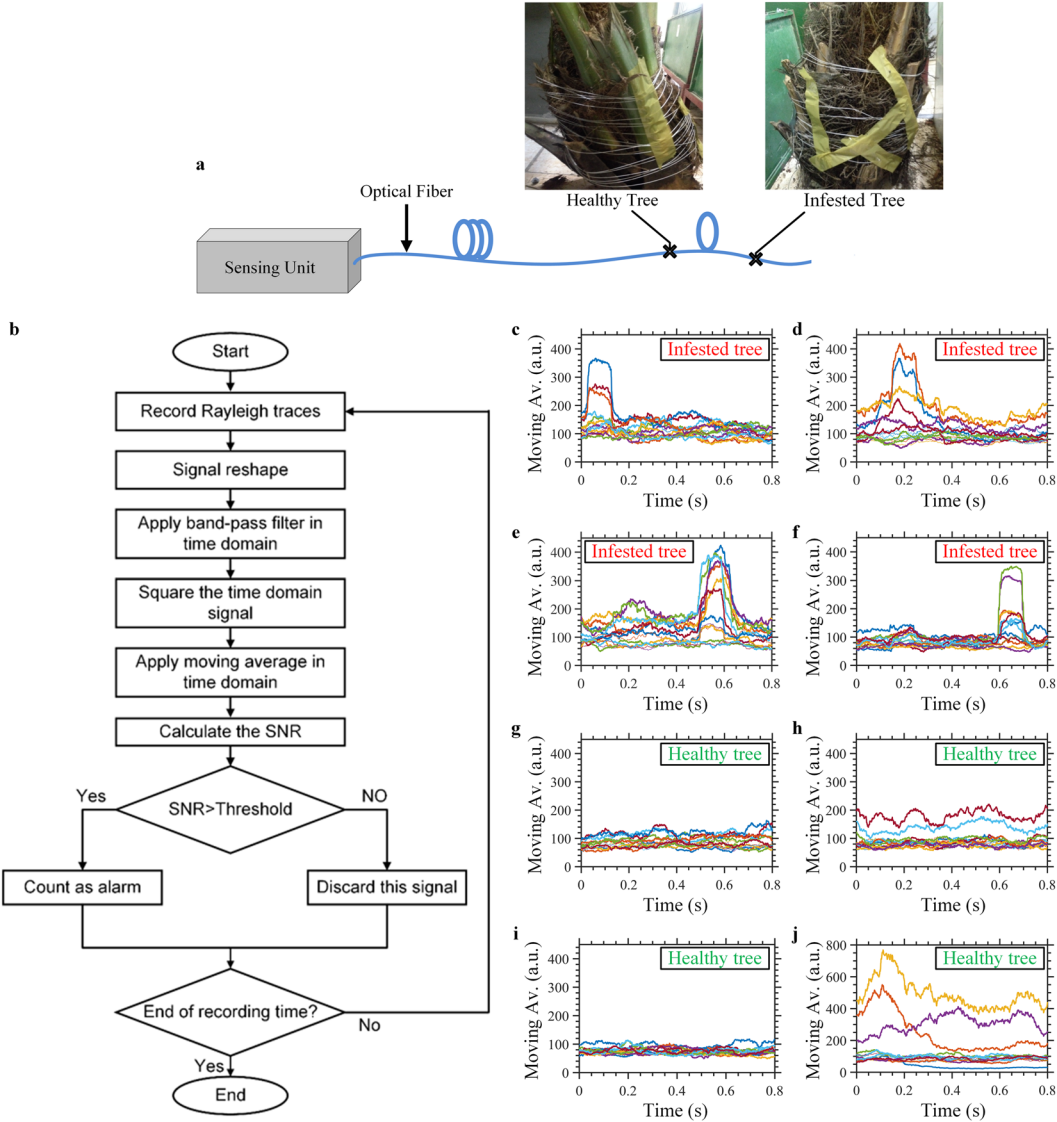
Distinguishing infested and healthy tree using optical fiber DAS. (**a**) Experimental configuration used to distinguish the infested from the healthy tree. (**b**) Flowchart of the algorithm used to detect the sound of weevil larvae. Representative examples of the moving average change, over time, for the sound signals collected from the infested tree (**c**–**f**) and the healthy tree (**g**–**j)**.

**Figure 5:**
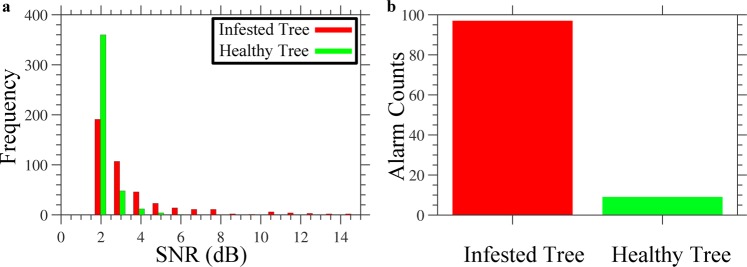
Distinguishing infested from healthy trees using optical fiber DAS. **(a**) Histogram of SNR values recorded for an infested and healthy tree. (**b**) Infestation alarm counts generated by our new sensor for an infested and healthy tree.

We repeated the recording of the larvae’s sound and the background noise, using the optical fiber DAS, instead of the voice-recording microphone. Near the end of the SMF, we wound a 10 m-fiber section around the same infested tree as that used for the former voice-recording microphone experiment. Based on the results obtained with the microphone, we applied a band-pass filter of [200–800 Hz] range to the time-domain signals collected by the optical fiber DAS in order to discard most of the low-frequency environmental noise, such as that from tree swinging^20^ and environmental thermal fluctuations^21^, as well as the high-frequency electronic noise of our system. While we were collecting the majority of the sound signal of the larvae. Two representative examples of the time-domain signals recorded using our DAS, after applying the band-pass filter, are shown in Fig. 3c,d; their corresponding power spectra are presented in Fig. 3e,f, respectively. For comparison purposes, using the same filtering band, and under similar experimental conditions, we recorded the time-domain signal and power spectrum of the background noise, using the optical fiber DAS (see example in Fig. 3g,h). Results show that the signal of the larvae signal is stronger than that of the background noise, in both time and frequency domains, meaning that our sensor has the ability to discover the presence of weevil larvae; however, to assess its reliability, it was necessary to perform additional statistical studies (demonstrated later in Figs. 4 and 5). Additionally, a more advanced signal processing technique may be needed to avoid the instantaneous noises that produce false alarms through interference with the real signals from the larvae. It is also important to clarify that the infested and healthy trees used in our experiments were all placed in a closed room with windows, not in an open-air farm. Consequently, these trees might be subjected to sound noises produced by birds flying around the room, and/or humans within the room, along with that from air flow, even if that flow was very weak. Distinguishing healthy from infested trees in open-air farms where the optical fiber might be subjected to harsh environmental noises produced by wind, rain, etc., is part of our plans for future work on this topic.

In this study, we also developed an algorithm to assess whether a palm tree is infested or healthy. We set our experimental configuration, as shown in Fig. 4a, where the sensing unit was connected to the same SMF of ~1.1 km length. Near the end of the fiber, we wound a 10 m-fiber section around a healthy tree and another identical fiber section around the previously used infested tree with ~12 days old larvae. The two trees were well-separated in space, so that the fiber’s length between them was ~40 m. This distance was more than enough for our sensor to recognize which tree emitted the recorded individual signals. Again, the two trees were placed in a closed room, not in an open-air farm (Fig. 4a). The detection algorithm is presented in the flowchart from Fig. 4b. We started with a continuous reading of the Rayleigh traces, for a 1-second period, which was limited by the memory size of the oscilloscope and computer specifications. We then reshaped this continuous recording Rayleigh traces into individual ones, such that each trace was the outcome of sending one pulse through the optical fiber. We then applied the band-pass filter of [200 Hz, 800 Hz] range on the time-domain signal of the individual points, along the optical fiber. Because of the noise introduced by the digital filter, we omitted the first and last 50 ms of the recorded data corrupted by the filter. Next, for each point along the optical fiber, we squared its time-domain signal and applied a moving average (with a 100 ms window) on the squared signal. There were four different scenarios that might impact on the results of the moving average: the first scenario was when our system recorded background noise without any instantaneous time-domain noisy spikes. In this case, the result of the moving average was almost of constant amplitude. The second case was when the background noise included some instantaneous noisy spikes. Since these spikes typically occurred within short time periods (i.e., ≪100 ms), the moving average results would still be of relatively constant amplitude. The third scenario occurred when the signal was captured while larvae were present and active. Based on data analysis, the optical fiber DAS typically did not record a continuous sound of larvae within a one-second time frame. This might be due to the fact that the eating behavior of the larvae was not continuous, and that the larvae instead ate in a discrete, on/off manner. Another possible reason might be that strength of the sound signal produced by the larvae was not constant therefore, when the sound signal was strong the optical fiber could record it; otherwise, the sound was weak to disturb the fiber refractive index. In this third scenario, the moving average result consisted of ups and downs. Finally, the last scenario, which rarely occurred, was when our sensor recorded a larvae’s sound signal continuously, during the one-second period, with almost the same strength, i.e., larvae did not stop eating and their signal reached the optical fiber with almost constant strength. In this case, the moving average result was of almost constantly a high value and behaved similarly as that obtained when recording the background noise, without ups and downs. After calculating the moving average (Fig. 4b), we determined a signal-to-noise ratio (SNR), defined as the ratio between the high-level and the low-level of the moving average. If the SNR was higher than a certain predetermined decision threshold, we counted this as a true alarm of larvae infestation; otherwise, we ignored it. Following the flowchart, when the experiment time ended, the algorithm stopped, and we could count the number of true alarms of infestation recorded during the experiment; otherwise, we repeated the process, starting by measuring 1-second periods of Rayleigh traces. The main drawback of this algorithm is when the fourth scenario occurred, i.e., our system continuously recorded a sound of the larvae that has a similar strength; in this case, the system could not generate a true alarm of infestation. However, even with such a drawback, the accuracy of our sensor is still acceptable, as will be seen later in Fig. 5b.

Figure 4c–f show four representative examples of the change in moving average, over time, for data collected from the infested tree. In our experiment, we used a 125 MHz oscilloscope sampling rate, which means that we sampled data every 0.8 m, along the optical fiber. Each graph in Fig. 4c–f includes 14 curves, one for each spatial point, which adequately covers the 10 m-section fiber wound around the tree. The SNR was calculated for all individual 14 curves and their maximum value was used for comparison with the decision threshold. Clearly, as shown in Fig. 4c–f, the moving average of the infested tree data includes ups and downs, as we expect. In contrast, Fig. 4g–j show the change of the moving average with time for the signals gathered using the healthy tree. The moving average results, in this case, indicate the presence of smooth fluctuations, in comparison with the infested tree. In some rare cases (Fig. 4j), the moving average of the healthy tree might include some ups and downs, and the corresponding SNR value exceeds the decision threshold to produce a false alarm of infestation.

For statistical analysis purposes, we kept running this experiment continuously, for two hours, while we calculated the SNR for both the infested and healthy trees. Figure 5a shows a histogram of the SNR values for the infested (red bar) and healthy (green bar) tree, when choosing 1 dB as a bin. Based on the results of this histogram, we selected 4 dB as the SNR decision threshold to determine whether the tree is infested or healthy. Using this decision-threshold value, our sensor provides 97 infestation alarm counts for the infested tree, versus 9 only for the healthy tree (Fig. 5b). These results confirm the ability of our developed sensor to distinguish the infested from the healthy tree, with a relatively high accuracy.

## Discussion

In this study, we developed an acoustic-sensing hardware and algorithm for monitoring and distinguishing infested from healthy trees, using an optical fiber DAS of 10 m spatial resolution and ~1.1 km fiber length. As reported in the literature, the sensing range of an optical fiber DAS can be easily extended to ~10 km, while keeping a 1 m spatial resolution^22^. Assuming the total separation between two consecutive trees and the fiber length wound around each tree is ~10 m, one sensing unit would theoretically be able to continuously monitor ~1000 palm trees. The current total cost of the sensing unit and 10 km fiber is ~US$37,000; ~US$37 monitoring cost per tree. In order to reduce the monitoring cost per tree, time-division-multiplexing (TDM)^23^ can be implemented based on our sensor. This could be performed via connecting multiple optical fibers to the sensing unit, through an optical switch. Alternatively, optical fibers could be permanently installed in palm farms while a sensing unit is shared among the farms at different time slots, which would significantly reduce the monitoring cost per tree.

The signal processing algorithm we adopted to identify infested trees is powerful and can be generalized for any event count analysis, using an optical fiber DAS. Typically, the SNR of optical fiber DAS is defined as the ratio between the peak-to-peak signal variations to that of the background noise, within a set time-domain^19^. Following this common definition, any noisy spike occurring at a tree location would provide a false infestation alarm. In contrast, our SNR definition significantly mitigates the trigger of false alarms. The moving average window, 100 ms in our analysis, can be tuned to even span the entire recording time period. However, as shown in Fig. 5a, the decision threshold should be carefully selected, based on the moving average window.

The significant advantage of our reported sensor over those found in literature is that the optical fiber DAS system offers distributed early detection of RPW. Within few minutes, an entire palm farm would be scanned using the reported sensor. Compared with the other RPW detection methods that rely on visual inspection^24^, x-ray^7^, acoustic probes^4^, dogs^8^, etc., our reported technique saves a lot of time and effort. However, in contrast to the invasive acoustic probes, the reported system would find challenges to distinguish the larvae’s sound from environmental noises, when using it in natural field environment. Fortunately, many successful techniques have been reported to recognize the RPW’s sound in noisy environments, when using acoustic detection methods. For instance, J. Pinhas *et al*. developed a mathematical method to automatically recognize acoustic activity of RPW in offshoots with a 98% average detection ratio^25^. Similarly, when using off-the-shelf recording devices, spectral signature of the RPW’s sound was utilized to detect RPW^26^. Additionally, R. W. Mankin *et al*. used spectral and spatial filtering, acoustic spectrum features, and acoustic temporal pattern features to identify the RPW’s sound from background noise^12,27–29^.

The developed sensor can concurrently be used to monitor the temperature to control farm fires, another major problem around the world. Distributed optical fiber temperature sensor can be designed using Brillouin optical time-domain reflectometry (BOTDR)^30^.

In summary, we reported a robust early detection method for RPW, using an optical fiber DAS. This new sensor aimed to detect the presence of ~12 days old weevil larvae in a palm tree. To do so, the sound spectrum signature of the weevil larvae was identified by recording the sound of the larvae, using a commercial voice-recording microphone. Using the sound spectrum signature obtained from measurements, a signal-processing algorithm was subsequently developed in order to analyze the data recorded by the sensor, and to distinguish infested from healthy trees. We found that, after a two-hour recording, our system provided 97 true infestation alarms for the infested tree, versus 9 false alarms for the healthy one. Once installed, the new sensing system could potentially offer a 24-7 monitoring for thousands of trees, over extended time periods. As a future work, we are planning to use the reported sensing system to distinguish healthy from infested trees in open-air farms.

## Methods

### Artificial infestation of a palm tree with red palm weevil

To artificially infest a palm tree with RPW, we firstly removed the fibers attached to the tree trunk. The trunk was then cut into two halves, along its length. Within one of the half-segment, we dug a hole to house the male and female adult RPWs. Next, the half-segments were rejoined to reconstruct the trunk which was kept at 27 ± 3 °C temperature and 65 ± 10% relative humidity. To avoid the adult RPWs from escaping the tree trunk, we firmly wound a steel wire around the trunk and along its length. The reconstructed trunk was opened after roughly a week to check that the female RPWs produced eggs. After which, the whole adult RPWs were removed from the tree, and we waited until the weevil larvae reached the desired age before running our sensing experiment.

### Calculating the signal-to-noise ratio

In our analysis, we defined the SNR as the ratio between the high-level and the low-level of the moving average. The moving average’s high level was calculated as the mean of the highest-valued 2000 points in the moving average. Considering the experimentally used 20 kHz repetition rate of injecting pulses into the optical fiber, the 2000 points were equivalent to the 100 ms window of the moving average. Similarly, the low-level of the moving average was determined as the mean of the lowest-valued 2000 points in the moving average. In our experiment, we used one infested tree and another healthy one. For statistical analysis purposes, the SNR values were continuously calculated over two hours for the two trees.
